# Bootstrap methods can help evaluate monitoring program performance to inform restoration as part of an adaptive management program

**DOI:** 10.7717/peerj.11378

**Published:** 2021-05-04

**Authors:** Jennifer F. Moore, William E. Pine III

**Affiliations:** Department of Wildlife Ecology and Conservation, University of Florida, Gainesville, FL, United States of America

**Keywords:** Monitoring program design, Adaptive restoration, Coastal restoration, Adaptive management, Oyster reef, Deepwater Horizon, Gulf of Mexico

## Abstract

The objective of many fish and wildlife restoration programs is to utilize management actions to change the state of a system. Because restoration programs are often expensive, iteratively assessing whether the restoration is having the desired outcome is a critical aspect of learning how to inform ongoing and sampling designs to evaluate proposed restoration programs. We provide an example of how we are using data resampling as part of an adaptive restoration process to test the effectiveness of a restoration action and associated monitoring program to restore the degraded Lone Cabbage oyster reef in Suwannee Sound, Florida in the northeast Gulf of Mexico. We use a resampling framework through simulations to inform the progress of the restoration efforts by examining the direction and magnitude of the differences in live oyster counts between restored and unrestored (wild) reefs over time. In addition, we evaluated the effort (number of sites sampled) needed to determine the effect of restoration to understand how many surveys should be conducted in subsequent sampling seasons. These efforts allow us to provide timely insight into the effectiveness of both our monitoring efforts and restoration strategy which is of critical importance not only to the restoration of Lone Cabbage Reef but to larger restoration efforts within the Gulf of Mexico as part of the consolidated *Deepwater Horizon* settlements and funded restoration efforts.

## Introduction

The goal of many fish and wildlife restoration programs is to alter a system from an undesired to desired state through the use of management actions. Restoration efforts are underway in the US Gulf of Mexico region across a diverse range of upland, inshore, and offshore habitats as part of the consolidated *Deepwater Horizon* settlements (National Academies of Sciences, Engineering, and Medicine, 2017). These restoration efforts occur at multiple spatial scales from individual projects at a single geographic location, the shoreline of a US state, or restoration at the scale of the entire Gulf of Mexico for wide ranging species or habitat. From a programmatic perspective, research and restoration efforts as part of the consolidated *Deepwater Horizon* settlements have been encouraged to follow an adaptive management framework (National Academies of Sciences, Engineering, and Medicine, 2017) as a process to integrate monitoring, evaluation of management actions, and address uncertainty in restoration decision making (NRC, 2014; NRDA Trustees, 2016). Adaptive management is a systematic approach for improving natural resource management by learning from management outcomes (Holling, 1978). In an active adaptive management framework (Walters & Holling, 1990) efforts are made to try and optimize short-term performance of the management action while developing insight into which competing system models are most useful for informing future management actions. In a restoration context, this can be described as taking actions in the short-term to trigger a desired response in the target species or ecosystem, while learning how to efficiently create these responses through the best restoration design in terms of materials, approach, and cost. Zedler (2017) reviewed adaptive management efforts in coastal systems with a restoration component and characterized these efforts as “adaptive restoration” programs. In this same review Zedler (2017) draws attention to the critical challenges facing decision makers in estuarine and coastal systems. These systems face uncertainties in system response both from landward inputs including changes in river discharge and nutrients and seaward inputs such as sea level rise and increasing frequency and intensity of storm events. This complicates detecting the restoration response by the system from the potentially large background noise in the system.

A challenge in using an adaptive management approach to inform restoration at different spatial and temporal scales is in repeatedly assessing target species responses to the management action (learning while restoring) to determine whether the restoration is having the desired effect, or if some sort of change in the restoration program is necessary. This differs from standard monitoring program design considerations in many fisheries and wildlife studies that may focus on the power to detect trends over time in some system indicator or response to a management action (Pregler et al., 2019; Wagner et al., 2013). As an example, Wagner et al. (2013) found that on average about 10 years or more of monitoring was required to detect less than a 5% change in a variety of biological metrics (i.e., fish abundance, biomass, density, CPE) with similar patterns for habitat indices.

In an adaptive restoration context where feedback loops exist to update and inform the restoration process (i.e., design of the restoration) while the restoration is ongoing, long-time lags (years to decades) in detecting a response to restoration are likely not acceptable (Zedler, 2017). This is because learning how a restoration could be improved after the restoration project has been completed is only useful if a subsequent restoration action can benefit from this information. In this case, learning becomes relevant to the scale of the restoration. At a local, individual restoration project level, learning needs to happen rapidly during the restoration process, for example, to inform decisions related to restoration design in order to improve the likelihood of the restoration having the desired outcome. In contrast at higher levels of project organization in space and time, cumulative incremental learning may be acceptable in reaching programmatic goals (i.e., restoration of resource at a coastline level) at the expense of optimizing the success of any one individual local project. Imagine example restoration projects of similar type and objectives at location A and B. In a passive restoration context, if a local restoration A does not trigger the desired response as predicted, then this knowledge of A not working can be used to inform the design and implementation of project B, increasing the likelihood of B triggering the desired response. In this way the costs of improving the likelihood of project B being successful is the cost of project A failing. However, if project A’s likelihood of failure can be detected earlier in the restoration process, then in an adaptive restoration context intervention in the restoration could be made while the restoration is ongoing. This would increase the likelihood of project A being successful while maximizing learning, but also increase the return on the restoration investment as there is now a higher likelihood of both projects A and B being successful. A critical aspect of the adaptive management process working is the ability to detect whether restoration project A is having the desired outcome which is dependent on performance of the monitoring program in place to track system responses to restoration.

We present an example from an ongoing restoration project on Lone Cabbage oyster reef in the Big Bend region of the Florida Gulf of Mexico coast. Our example is a local, individual project level, in-progress, adaptive restoration case history where we identify significant uncertainties to overcome related to oyster reef restoration and demonstrate how using iterative analyses of data as they are collected can be used to assess the “informativeness” of a restoration program. This example differs from traditional power analyses in several ways. By design our approach is “post-hoc” in that it is using data from each of our sampling events after these data have been collected, to determine whether these data are sufficient to detect an effect and how the effect size varies over time. In contrast a traditional power analysis is often prospective, asking what sample sizes would be needed to detect a hypothesized effect based on some critical level of significance.

## Materials & Methods

### Study site

**Figure 1:**
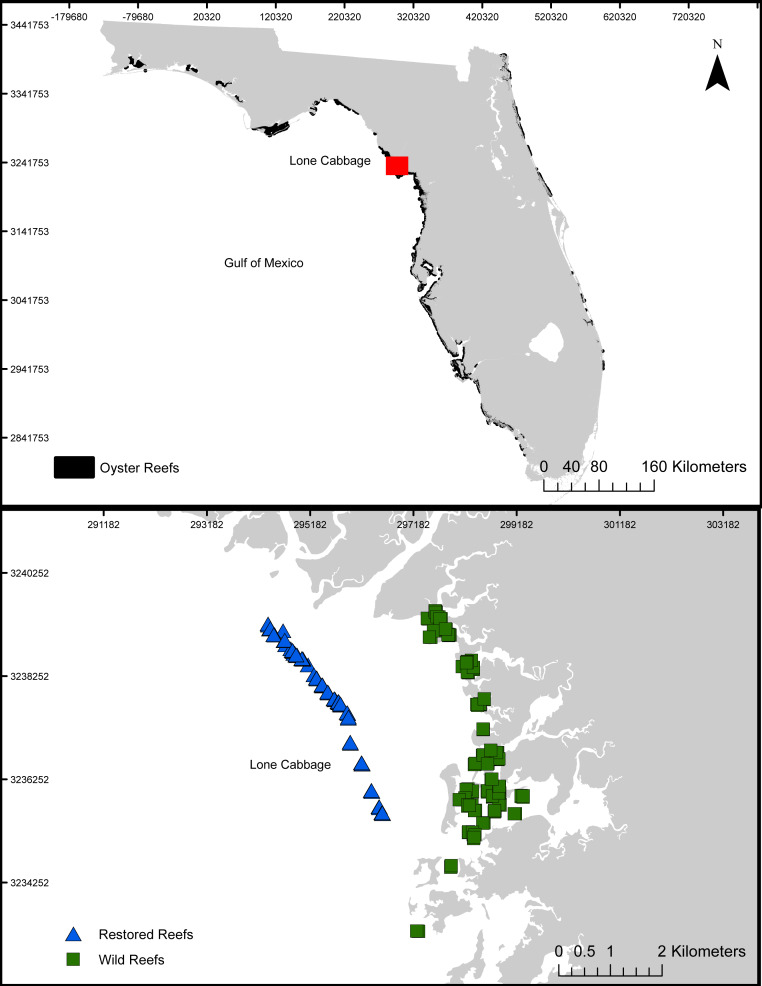
Lone Cabbage Reef study area. The location of Lone Cabbage Reef within the Gulf of Mexico including the restored and unrestored (wild) reefs.

Lone Cabbage oyster reef is a chain of intertidal oyster reefs located approximately 3 km south of the East Pass of the Suwannee River and 12 km north of the town of Cedar Key, Florida (Fig. 1). Oyster reefs in this region commonly form linear chains parallel to the shoreline and provide multiple ecosystem services ranging from fisheries, protection of shoreline features, and promotion of estuarine conditions (Kaplan et al., 2016). In recent decades, Lone Cabbage Reef has degraded in terms of oyster coverage and elevation of the reef surface (Frederick et al., 2016; Moore et al., 2020; Seavey et al., 2011), potentially reducing the effectiveness of this reef complex in providing both local (e.g., oyster habitat for fish and wildlife) and large-scale (e.g., promotion of estuarine conditions; Kaplan et al., 2016) ecosystem benefits and fishery harvests. Since multiple lines of evidence have identified substrate as a limiting resource (Frederick et al., 2016), our restoration approach uses large, quarried limestone rock (average size 52 × 34 × 33 cm) to rebuild the elevation and historic footprint of Lone Cabbage reef identified from surveys completed in the 1800’s (Raabe, Streck & Stumpf, 2004). Oyster monitoring efforts focus on assessing restoration goals of increases in Lone Cabbage oyster population (counts from transects) on restored oyster bars compared to adjacent extant unrestored (wild) oyster bars, which may be declining (Moore et al., 2020).

### Oyster monitoring

We monitor intertidal oyster populations along restored and unrestored (wild) reefs using line transect methods to capture the potentially high variation in oyster populations across the surface of the reef (Krebs, 1999; see application in (Moore et al., 2020). Individual reefs within the restored and unrestored areas are chosen at random from all possible reefs in the study area based on available GIS layers (https://geodata.myfwc.com/datasets/oyster-beds-in-florida). Possible line transects along each randomly chosen reef are then identified, with transects crossing the centerline of the reef perpendicular to its longest axis. Transects sampled are then chosen at random from this list of possible transects. The start and end points are then recorded in the field with a GPS in the field with <1 m accuracy and welded rebar frames are temporarily placed at the start and end to delineate the transect. All transects are assessed in the field to verify sampling is possible with regards to tidal height and other factors. Pairs of strings are tied between the frames to create a long, thin, belt transect (Krebs, 1999). Observers then walk along these transects counting the number of both live and dead oysters in each 2.5 m interval of the transect visually using hand-held tally counters. A subset of these transects are counted by 2 observers to assess variation among the observers. All observers are trained using a surrogate artificial oyster reef with known numbers of oysters prior to conducting field sampling. Field experiments were approved by the Florida Department for Environmental Protection.

### Assessing monitoring program performance to inform restoration

To assess the performance of our monitoring data to inform our ongoing restoration efforts, we developed a framework of resampling data collected during the first 18 months of our monitoring effort post-construction to assess the “informativeness” (see Bolker, 2008; Johnson et al., 2015) of our oyster counts to detecting a response to the restoration. Using count data for many species of fish and invertebrate species as a response variable can be difficult because of high annual variation in survival, recruitment, and capture probability (see examples in George, Baldigo & Stich, 2019; Gillespie & Vincent, 2019; Gwinn et al., 2016; Hayes et al., 2019; Hilderbrand & Kazyak, 2017; Miner et al., 2018; Wagner et al., 2013; Wagner et al., 2014) which could mask restoration effects. This is an area of significant concern because failure to detect whether a restoration action has the desired effect in a timely way significantly limits opportunities actively to inform ongoing restoration actions.

While we recognize the immediate post-construction period may be a period of transient dynamics in the Lone Cabbage Reef oyster population, capturing this variability in our analyses is an essential part of our short and long-term adaptive management learning process. Our concern was that variation as a result of inherent short-term instability in our key response variable (live oyster counts) during the transitory period post-construction would preclude our ability to evaluate, learn, and adjust our restoration program if necessary. This is important at both the project, and at larger (program) scales as this restoration project is relatively early in the project scope (year 3 of 8).

Following guidelines in Bolker (2008), Johnson et al. (2015) and Kain, Bolker & McCoy (2015) we evaluated power to detect significant differences in oyster counts between restored (rock addition) and unrestored (no rock, i.e., wild reefs) reefs. This is done where we: (1) resample 5000 data sets based on observed count data from restored and unrestored sites (i.e., sample with replacement separately within restored and unrestored sites), (2) fit a generalized linear model (GLM) with a negative binomial error distribution to each data set to test whether there is an effect of adding rocks on oyster live counts (i.e., examine whether the slope (beta) coefficient is significant), (3) and examine the ratio between predicted live oyster counts for unrestored sites (randomly selected wild oyster bars that have no history of restoration) to restored sites (the degraded oyster bars that have been restored via addition of rocks) to show the magnitude of difference between the restored and unrestored sites (see Appendix S1 for R code). For our GLM, we fit a model with live oyster counts as the response variable and a dummy variable for restored/unrestored reefs as the predictor variable. We included transect length as an offset to account for the differences in transect length between sites (Moore et al., 2020). We compared these results for each of our post-construction monitoring periods. In this example, period 1 refers to the winter of 2018–2019 (*n* = 61 oyster bars) and period 2 refers to the winter of 2019-2020 (*n* = 45 oyster bars). By using resampled data, we can better understand how likely we are to fail to detect a response when one is occurring, given the variation in our response data in years following construction. In general, resampling is advantageous in this situation over traditional power analyses because no assumptions need to be made about the distribution of the data within each stratum. In addition, using resampling allows us to quantify the uncertainty of an estimate, in our case assessing the direction (increase or decrease as a positive or negative sign) and magnitude (measured by the beta coefficients) of the change in oyster counts over time. In an adaptive management context this is important because it helps us to determine how likely we are to detect whether the restoration is triggering the desired response in both direction and magnitude.

We then examined the change in the ratio of predicted live oyster counts for unrestored sites to restored sites between period 1 and 2 to estimate the number of years it would take for restored reefs to become more similar to unrestored, wild reefs in terms of live oyster counts, if the same trajectory is followed. This metric is complicated because wild reefs are in decline (Moore et al., 2020; Seavey et al., 2011). And lastly, we evaluated the approximate effort (number of sites sampled) needed to determine the effect of restoration on live oyster counts (measured as the sign and magnitude of the beta values) to understand how much survey effort is necessary in the next years of sampling.

## Results

Using replicate simulated data sets (*n* = 5,000) based on field observations recorded 6 months post-restoration (period 1, winter 2018–2019), our GLM results showed that nearly all simulated data sets (4,941/5,000) resulted in higher live oyster counts for unrestored vs. restored sites (i.e., 4,941/5,000 of the simulations had beta coefficients <0), suggesting there was about a 99% chance our monitoring efforts are sufficient to determine whether there is an effect of the restoration. The 95% range in slope (Beta) coefficients was −1.064 to −0.045 which suggests that, when controlled for effort, unrestored oyster bars will have between 5 and 106% more oysters than restored oyster bars.

We repeated these analyses with data 18 months post-restoration (period 2, winter 2019–2020). Again, most simulated data sets (4,984/5,000) suggested higher live oyster counts in unrestored than restored sites (i.e., 4,984/5,000 of the simulations had beta coefficients <0). The 95% range of Beta values was −0.757 to −0.120 which suggests that 18 months post restoration unrestored oyster bars will have between 12 and 76% more oysters than restored oyster bars.

**Figure 2:**
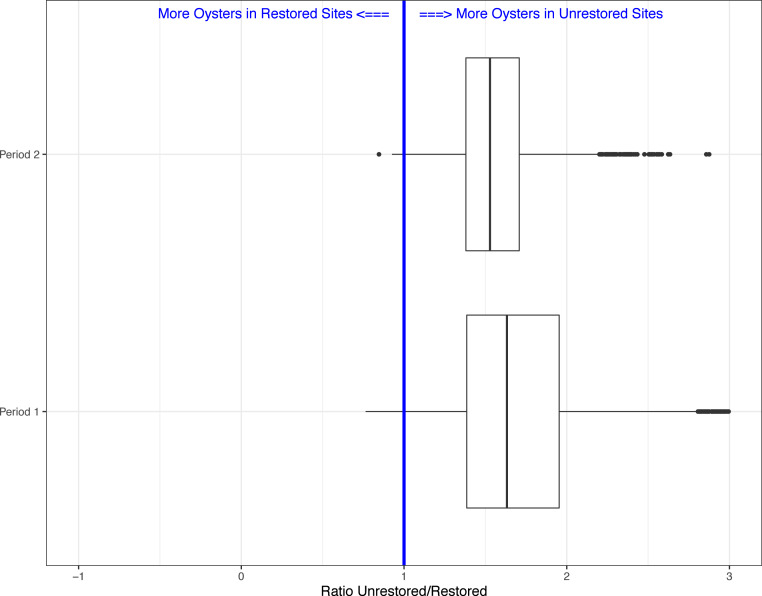
Boxplots showing the ratio of live oyster counts between the unrestored (no rocks) and restored (rocks) sites. Boxes show the ratio from the 5,000 simulations for period 1 (Winter 2018–2019) and period 2 (Winter 2019–2020).

Between period 1 and 2, we found that the ratio of live oyster counts between unrestored and restored sites changed from on average 1.73 (SE = 0.0068; i.e., on average 73% more oysters predicted on unrestored bars), to 1.56 (SE = 0.0036), or a 17% increase in predicted number of oysters on restored sites relative to unrestored sites after 18 months (Fig. 2). If we assume that restored oyster bars will continue to accumulate oysters at a similar level in future years, then we expect live oyster counts to be equal between unrestored and restored sites in about 5.5 years post construction.

**Figure 3:**
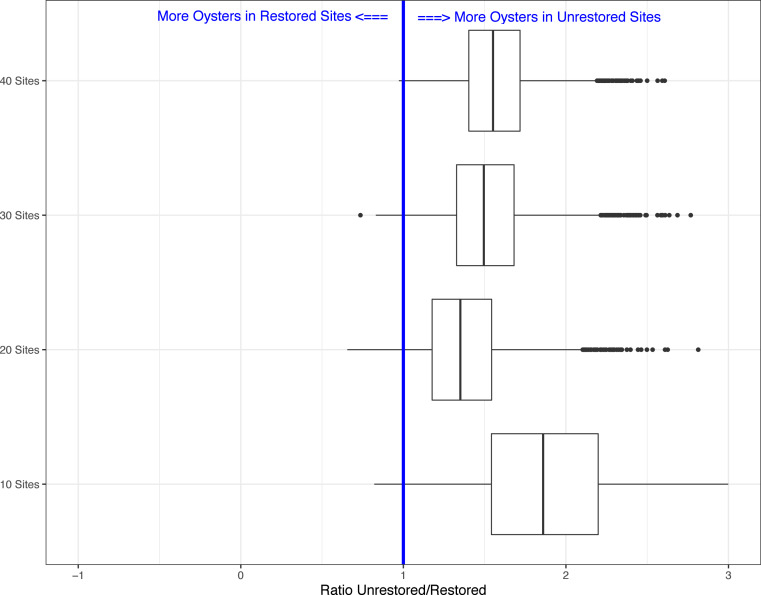
Boxplots showing the ratio of live oyster counts between the unrestored (no rocks) and restored (rocks) sites. Boxes show ratio from the 5,000 simulations for a subset of 10 sites, 20 sites, 30 sites, or 40 sites of the total number of sites from period 2 (winter 2019–2020).

Lastly, because our field sampling is limited to a few tidal cycles per year and sampling costs are high, we examined whether we could detect differences in live oyster counts between unrestored and restored sites at a lower sampling effort than what we conducted in periods 1 and 2. In period 2, 10 restored sites (of multiple transects, average sum of transect lengths about 34-m) and 35 unrestored sites (average sum of transect lengths about 144-m) were sampled, for a total of 45 sites. We created 5000 simulated data sets using a subset of the data collected in period 2. We examined four different simulations assuming several alternative sampling regimes: 5 restored and unrestored sites (10 total), 10 restored and 10 unrestored sites (20 total), 10 restored and 20 unrestored sites (30 total), and 10 restored and 30 unrestored sites (40 total). We used 10 restored sites as the maximum since that was the total surveyed during period 2. Using all sample sizes from 10 surveyed reefs to 45 (total surveyed during period 2), we found that for 10 sites the average Beta value was −0.607(range −1.478 to 0.197). The range of values declined as the number of sites increased (20 sites: range −1.035 to 0.422; 30 sites: range −1.018 to 0.306; 40 sites: range −0.958 to 0.028) with negative values suggesting more live oysters on unrestored vs. restored reefs. This was found in a minimum of 93% of simulations depending on sample size (Figs. 2 and 3). Sampling as few as five restored and five unrestored sites in period 20 is likely to detect whether differences exist between live oyster counts on restored and unrestored reefs in 98% of our simulations.

## Discussion

The restoration of Lone Cabbage oyster reef has been improved by using an adaptive management framework to guide restoration actions. Using an adaptive approach necessitated the development of a high-resolution monitoring program to separate restoration response, from the background noise inherent in many natural resource monitoring programs. These types of monitoring efforts have been identified as a critical component to a successful adaptive management project (Walters, 2007). Iteratively analyzing data from both field collections and simulations based on these field data have also helped us to clarify our thinking related to evaluating restoration in that we are not only testing “do restored and unrestored sites differ” but also the goal of our restoration (assessed by monitoring) is to document: (1) whether the restored and unrestored sites are becoming more similar, and (2) are the restored reefs more resilient to collapse? These simulations suggest that our monitoring efforts as currently implemented are likely adequate for tracking counts of live oysters on restored and unrestored oyster bars, even with the high variation in counts observed. This result was true even if only 5 restored and 5 unrestored sites were sampled. Sample sizes less than 5 sites within each treatment would likely be less ideal because the results would be completely dependent on the randomly chosen reefs (i.e., if two low density reefs are chosen in the unrestored area but two high density reefs in the restored then the live oyster counts for restored reefs would be higher than unrestored and the beta coefficient would be positive). Having a minimum of 5 sites within each category (restored and unrestored) appears to be the minimum number of samples that can be taken to capture the underling variability in oyster counts in the line transect data from field efforts. In general, we have found that counts which follow a negative binomial distribution, as we have found for counts of live oysters on reefs in the Big Bend region of Florida (Moore et al., 2020), require higher sample sizes to attain the same confidence limits than data which follow a Poisson or normal distribution (Krebs, 1999). Our results also suggest that, 1.5 years post-restoration, the restored oyster reef is on a trajectory to become similar to the unrestored, wild oyster reef (Fig. 2), and that the likely timeframe to convergence is about 5 years. A larger context for this adaptive restoration is that since 2010, we have documented declines in counts of live oysters on intertidal reefs (Moore et al., 2020) over a larger spatial scale than just Lone Cabbage reef. One key aspect of the restoration to monitor over time is whether the restored reefs will continue to accumulate oysters until a carrying capacity is reached, and once reached, if oyster counts will persist at this level or if the restored oyster bars will begin to show declines in counts of live oysters as has been observed on the wild bars. A key uncertainty, and one of the motivations for this restoration and research, is whether the restored oyster reefs have a higher potential or “floor” in terms of oyster abundance, than the unrestored, wild bars. This floor is essentially a baseline oyster population size that might be a result of increased live oyster or shell densities sustainable on the new substrate, and/or a result of oyster populations being more resilient over time to mortality events because of the added substrate. This is because of the addition of the rock during restoration, oyster abundance on the restored bars will not decline to the point of reaching a tipping point to transition to the persistent, highly resilient degraded oyster reef state. These differences in potential would arise because the rock is more permanent than dead shell and provides a stable nucleation site for oyster spat to settle assuming they are available (Frederick et al., 2016).

Our experience with the highly variable live oyster counts has also motivated us to assess whether other response variables such as oyster shell biomass per unit area (Pine et al., 2015; Zacherl, Moreno & Crossen, 2015) or oyster meat biomass per unit area (Colden, Latour & Lipcius, 2017) may be less variable, and thus more sensitive, to treatment effects and as such a better response variable. This is because shell or meat biomass per area would be the sum of the individual measurements for an area of reef (i.e., m^2^), for example the sum of meat biomass for a few large oysters or many small oysters may be less variable than the counts of individual oysters. Because our field sampling days are limited by tidal height, we can use our simulation model to assess tradeoffs in sampling effort such as whether to reduce sampling days from the monitoring program and allocate those days to research efforts such as assessing other response metrics. This helps us critically examine our main research question—is our restoration strategy effective or are we simply wasting a lot of time, resources, and learning opportunities through an uninformative field-sampling program? At present, it appears winter-based monitoring efforts are adequate to evaluate our restoration action.

## Conclusions

Restoration projects have inherent design and analytical challenges that differ from other types of monitoring programs (Carpenter et al., 1989; Conner et al., 2016; Stewart-Oaten, Bence & Osenberg, 1992). From a design perspective, restoration projects often fail basic best classical experimental design practices because (1) site selection is not random, (2) replication is often challenging because of costs and logistical constraints, and (3) defining a control can be challenging especially for large-scale projects. From an analytical perspective, the choice of the response metric used to assess restoration effectiveness also has major implications (Osenberg et al., 1994), such as the sensitivity of the response to the restoration action or being able to separate the response from background noise inherent in many biological data such as survival rates or counts. The use of control and manipulated sites is common in many restoration projects with the basic idea that the control site provides insight into how the restored site would have responded if restoration had not taken place. In our demonstrated application, because the un-restored sites may also be changing (Moore et al., 2020) we use our response data from the restoration site to assess both the effect (whether the beta terms <0) and how the effect size varies (the value of the betas). While this deviates from some recommendations in Wagner et al. (2013) review of power analyses in some types of monitoring programs, our approach provides a framework for addressing the direction, rate, and magnitude of oyster population responses to restoration actions within a time frame that allows us to inform restoration within this, and related restoration projects occurring within the Gulf of Mexico and elsewhere.

##  Supplemental Information

10.7717/peerj.11378/supp-1 Supplemental Information 1 Resampling simulation analysis R code and explanation We present the steps for resampling data through simulations to inform the progress of the restoration efforts.

10.7717/peerj.11378/supp-2 Supplemental Information 2 Live oyster counts from Period 1 (Winter 2018-2019) Raw data, which is the input file for the Rmarkdown code provided.

## Additional Information and Declarations

### Field Study Permissions

The following information was supplied relating to field study approvals (i.e., approving body and any reference numbers): Field experiments were approved by the Florida Department for Environmental Protection.

### Data Availability

The following information was supplied regarding data availability: An R markdown file explaining the R code for the resampling framework for assessing the differences in live oyster counts between the restored and unrestored oyster reefs is available as the Supplementary File.
